# Minor salivary gland biopsy in Sjögren’s syndrome: A review and introduction of a new tool to ease the procedure

**DOI:** 10.4317/medoral.19131

**Published:** 2013-08-29

**Authors:** Pablo Varela-Centelles, Juan M. Seoane-Romero, Mariña Sánchez-Sánchez, Antonio González-Mosquera, Pedro Diz-Dios, Juan Seoane

**Affiliations:** 1 Stomatology Department. School of Medicine and Dentistry. University of Santiago de Compostela. Santiago de Compostela. A Coruña. Spain

## Abstract

Objectives: To review the existing techniques for minor salivary gland biopsy (MSGB) in the lip and to suggest a new approach to ease the procedure and reduce post-operative complications. Study Design: A comprehensive literature review and a descriptive study of a new surgical technique. Results: Diverse incisions have been suggested for MSGB with different designs (ellipse, circular, linear), different directions (parallel, oblique, vertical) and a wide range of lengths (from 1 mm up to 3 cm), but no comparative studies supporting the advantages of a particular type of incision over the others could be retrieved. A variety of features of the existing techniques for MSGB are linked to undesired events and surgical complications which could be minimized by modifying certain aspects of these procedures. The technique described, together with the use of the S forceps, represents a significant improvement over the already described chalazion forceps because it allows for a better access and positioning of the lower lip, improves the ergonomic conditions of the assistant, and facilitates the identification of lip areas with more superficial gland lobules. Conclusions: The suggested approach for lip MSGB includes a specifically designed instrument whose performance during lip biopsy may contribute to minimize post-operative complications.

** Key words:**Sjögren’s syndrome, diagnosis, minor salivary gland biopsy, surgical technique, lower lip.

## Introduction

The Sjögren’s syndrome (SS) is an autoimmune exocrine disorder with signs and symptoms of dry mouth and keratoconjunctivitis sicca, which may sometimes display a wide range of systemic, non-glandular alterations (1,2). The prevalence of this syndrome has been estimated to range between 0.5% and 1% (3), with a female:male ratio of about 9:1 (1-3).

Histopathology in minor salivary gland (presence of focal lymphocytic sialadenitis with a focus score ≥1) is one out of the six diagnostic criteria set in the revised international classification for Sjögren’s Syndrome (2) for diagnosis of SS. It has recently become more important because of the consensus in considering only objective criteria to define a SS case, which has to meet at least 2 of the following 3 findings: 1. Positivity serum anti-SSA and/or SSB; 2. Ocular staining score >3; and 3. Presence of focal lymphocytic sialadenitis with a focus score >1 per 4 mm2 of glandular tissue (2).

A systematic review on minor salivary gland biopsy (MSGB) has proved diagnostic value for SS with high specificity (X±SD= 88.1±11.7) and sensitivity (X±SD= 78.8±11.2), as well as diagnostic confidence in terms of positive (X±SD= 87.6±9.5) and negative (X±SD= 79.0±16.9) predictive values (3). These results make this technique particularly useful for patients suspicious for SS with inconclusive clinical findings (4). MSGB may also contribute to diagnosis of amyloidosis, sarcoidosis, and confirmation of neonatal hemochromatosis (3,5,6).

Despite the different surgical approaches suggested for MSGB (use of chalazion forceps for tissue stabilization, usage of scalpel vs. punch, different incision sizes, and need or not for suturing), both immediate and mediate complications are continuously described in the literature, being the most relevant a long-lasting lower lip numbness occurring in up to 6% of MSGB procedures (7). These events support the need for a review of the technique to reduce morbidity. In this sense, we suggest the use of a specifically designed forceps for lip biopsy in SS patients that improves tissue stabilization, eases the procedure, and reduces complications.

## Material and Methods

**Figure 1:**
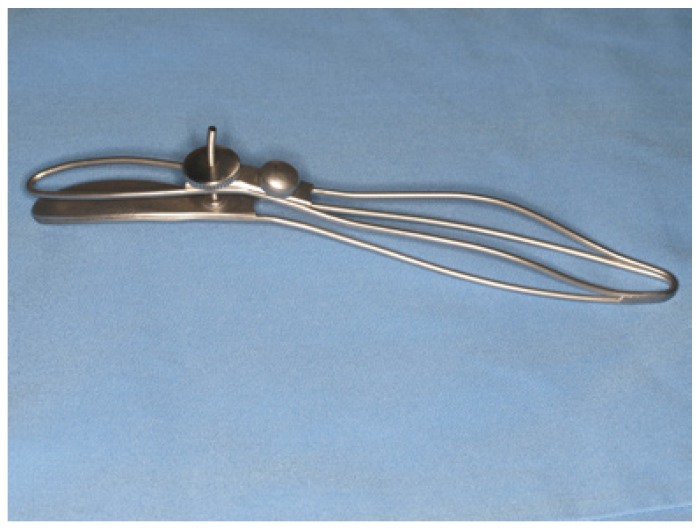
“S” forceps.

The materials required for this technique include a syringe for intraoral local anaesthesia, scalpel with a No. 15 blade, non-toothed Adson forceps, 4/0 braided silk suture, and the “S” forceps for biopsy (OEPM nº 201200158) (Fig. 1). This is a 18.5 cm long forceps with a fenestrated active end (5 cm2). Both the fenestrated area (longitudinal to the axis of the forceps) and its wide size are conceived to provide an ample surgical field. The non-fenestrated blade of the forceps is slightly convex in shape to facilitate herniation of minor salivary gland lobules. There is a screw in the shank for adjustment of the space between the blades, thus permitting a variable and controlled pressure over the soft tissues during the surgical procedure. The handles of the forceps are at an angle with the blades to help traction and visibility of the surgical field. This angle also permits the forceps to work as a surgical separator improving accessibility by means of a traction-separation movement.

Technique

**Figure 2:**
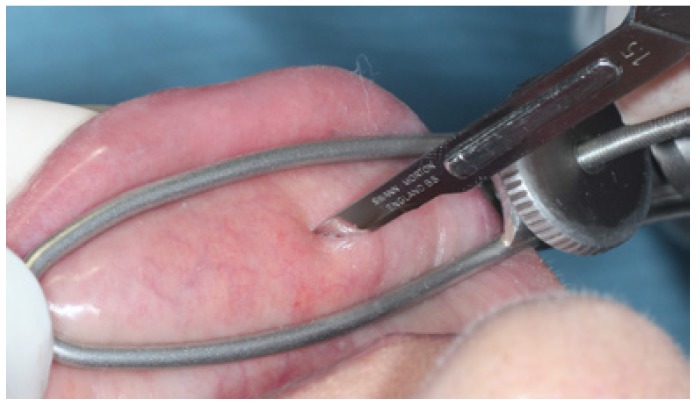
“S” forceps in use.

The biopsy site should be selected from the inner side of the lower lip, rich in minor salivary glands, avoiding the midline area due to its lesser content of glandular component (Fig. 2).

Local anesthesia is performed by perilesional infiltration or blockage of the mental nerve. Once anesthesia is achieved, the whole lower lip is stabilized using the S forceps, and the biopsy site selected taking advantage of the forceps design which forces the gland lobules to protrude through the fenestrated blade.

**Figure 3:**
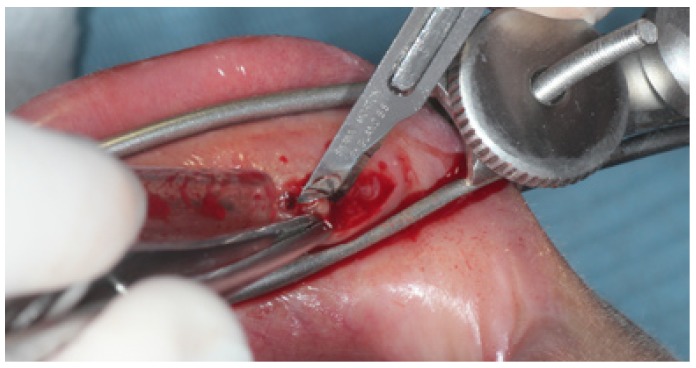
Excision of a minor salivary gland.

A horizontal linear incision of about 1 cm to 1.5 cm is performed away from the midline, combined with a blunt dissection of the borders of the wound. At this stage, the lobules are herniated towards the surface of the wound pushed by the non-fenestrared, convex, blade of the forceps (Fig. 3). Five to seven lobules can now be gently removed using the Adson tweezers and introduced into an abundant fixing solution (at least ten fold the volume of the tissue sampled). The wound is then sutured with interrupted single sutures. Use of magnification is recommended when performing the technique in order to identify superficial nerves and vessels and to diminish surgical morbidity.

Observations about the technique

MSGB of the lip is a key diagnostic tool for the diagnosis of systemic disorders and particularly of SS.

**Figure 4:**
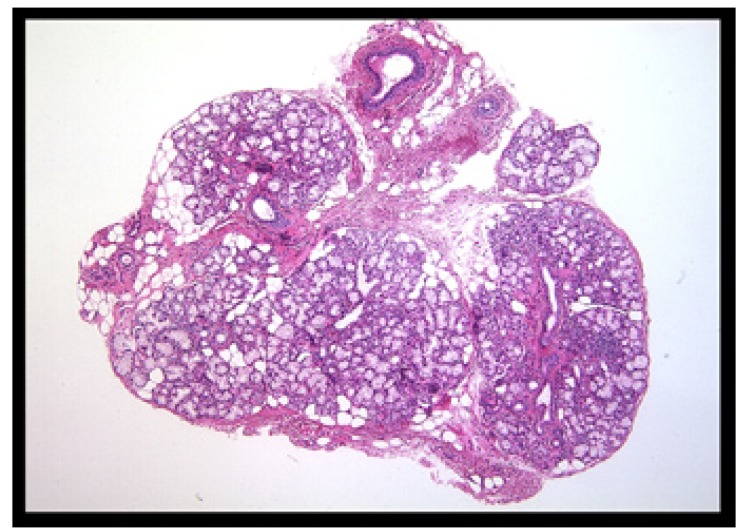
Minor salivary gland (H&E x10).

The technique described above, together with the use of the S forceps, represents a significant improvement over the already described chalazion forceps because it allows for a better access and positioning of the lower lip, improves the ergonomic conditions of the assistant, and facilitates the identification of lip areas with more superficial gland lobules. It also permits a better bleeding control during surgery, an enhanced visualization of vessel and nerve endings, reduces the surgical time, and provides non-artefacted lobules for pathological analysis (Fig. 4).

## Discussion

Techniques and complications in MSGB

Despite there is a wide agreement on avoiding the glandular-free zone in the centre of the lower lip, it seems to exist a remarkable lack of standardisation of the MSGB technique when aimed at obtaining at least five glandular lobules for the diagnosis of SS (8).

Different incisions have been suggested with different designs (ellipse, circular, linear), different directions (parallel, oblique, vertical) and a wide range of lengths (from 1 mm up to 3 cm), but no comparative studies supporting the advantages of a particular type of incision over the others could be retrieved (9-19).

Most frequent immediate surgical complications include intra- and post-operative bleeding (9,11). Pain, inflammation, wound infection, suture dehiscence, and cheloid scars are described as mediate complications of glandular biopsy (7,9-15), but the so-called “disorders of lip sensitivity” are the most frequently reported complication (18,19), occurring in up to 11% of cases in large series (12). This finding has discouraged the use of a punch for MSGB because it removes lip mucosa together with the attached gland, and favoured techniques that permit identification and avoidance of sensory nerve endings (16). These complications may well justify that only patients in a community setting with negative results for anti-RO/la antibodies would be referred for MSGB (20).

Lip stabilization devices

In this sense, some authors have suggested the use of chalazion forceps, employed by ophtalmologists during chalazion exeresis, to ease biopsy of minor salivary gland from mobile lip tissue, as it permits tissue stabilization and to work under ischemic conditions (6,7). However, this instrument was originally designed for ophthalmology and has a number of shortcomings for oral use: the handles of the chalazion forceps are small-sized to allow finger control and are placed perpendicular to the main axis of the blades; this forces the assistant’s hand to work on an uncomfortable position, too near to the surgical field. The size of the fenestration also limits the incision design, particularly when undertaking minimally invasive techniques with multiple 2 mm incisions along the inner face of the lower lip (13,18,19). An improved chalazion forceps was introduced by López-Jornet et al. (21): this forceps was larger than the original (20 cm.) and its active end provided a constant pressure of 1Kg/cm2 on the tissues exerted by means of two flat plates (one of them with a round opening, sized 1.7 cm diameter). This design eases lip stabilization by the assistant, but it is impossible to graduate the pressure on the lip tissue and the fenestrated blade provides a reduced surgical field.

On the other hand, the forceps we suggest for MSGB, besides permitting a controlled pressure adapted to the surgical time and to the features of the lip of the patient (macrochelia, etc.), allows a more ergonomic hand grasp in such a way that keeps the assistant’s hand away from the working area without disturbing the surgeon. Moreover, the width of the fenestrated blade in this forceps conditions neither the design nor the size of the incisions as well as permits minimally invasive techniques, where a wide surgical field is required to harvest glandular tissue all over th inner side of the lower lip (18).

## Conclusion

This forceps stabilizes lip tissues, avoids excessive intra-operative bleeding, permits better visibility of the surgical field, allows improved selection of tissue samples for pathological analysis and has a potential to minimize the morbidity related to iatrogenic nerve lesions.
